# Ptsd among a sample of french students: A misdiagnosed condition with many implications. preliminary results

**DOI:** 10.1192/j.eurpsy.2021.1201

**Published:** 2021-08-13

**Authors:** G. Païs, L. Romo, E. Tarade, S. Vigne, L. Cassé, G. Chaudoye, S. Julien Sweerts, R. Zebdi, D. Fouques

**Affiliations:** 1 Ea 4430 Clipsyd, Department Of Psychology, Université Paris Nanterre, Nanterre, France; 2 C2s Ea 6291, France, Université de Reims Champagne Ardenne, Reims, France

**Keywords:** students, ptsd, Experiential Avoidance, rumination

## Abstract

**Introduction:**

Students often suffer from stress, anxiety and depression (Saleh et al., 2019). However, research on PTSD is scarce among this population.

**Objectives:**

We therefore wanted to explore the presence of PTSD and other psychopathological and psychological variables in this population.

**Methods:**

We recruited 70 students -150 still planned- (22 years old, 70% women, 84.3% in the third year undergraduate) who filled out questionnaires at the university, after ethic committee’s approval.

**Results:**

31.2% show PCL-5 scores in favor of a PTSD. The most frequently mentioned traumatic events (direct exposure) are physical assaults (49.3%), transport accidents (29.4%) and unwanted sexual experiences (23.2%). According to the Mann-Withney U test, if they do not differ from students without PTSD in the number of traumatic events encountered (LEC 5 ns), they show more stress, anxiety and depression (p <.02), more dissociative symptoms (p <.04), less social support available (p = .048), a gap between the importance given to studies as a value and action directed towards this value (p = .002), idem for leisure activities (p =.035), and more rumination (p <.001) and more experiential avoidance (p <. 001). These two latter appear to be powerful processes involved in PTSD, as the PCL5 score is 37% explained by avoidance and rumination, according to linear regression.

**Conclusions:**

These preliminary results tend to show that PTSD should be investigated in students and seems to be linked to higher emotional difficulties, lower academic and social involvement. Rumination and avoidance could be an important therapeutic target.

